# *Candida auris*–Associated Candidemia, South Africa

**DOI:** 10.3201/eid2007.131765

**Published:** 2014-07

**Authors:** Rindidzani E. Magobo, Craig Corcoran, Sharona Seetharam, Nelesh P. Govender

**Affiliations:** National Institute for Communicable Diseases, Johannesburg, South Africa (R.E. Magobo, N.P. Govender);; National Health Laboratory Service, Johannesburg, South Africa (S. Seetharam);; University of the Witwatersrand, Johannesburg (S. Seetharam, N.P. Govender);; Ampath National Reference Laboratory, Pretoria, South Africa (C. Corcoran)

**Keywords:** Candida auris, South Africa, candidemia, fungal

**To the Editor:** We noted the report by Chowdhary et al. (*1*) and report *Candida auris* as a causative agent of candidemia in South Africa, with an estimated prevalence of 0.3% (N.P. Govender et al., unpub. data). First isolated in 2009, *C. auris* is an emerging species associated with clinical disease (*2*–*6*). We analyzed 4 isolates submitted to the National Institute for Communicable Diseases (Johannesburg, South Africa) from 4 patients with candidemia who had been admitted to different public- and private-sector hospitals from October 2012 through October 2013.

Identification of the isolates was undertaken by using ChromAgar *Candida* medium (Mast Diagnostics, Merseyside, UK), Vitek-2 YST (bioMérieux, Marcy ľEtoile, France), API 20C AUX (bioMérieux), and sequencing of internal transcribed spacer (ITS) and D1/D2 domains of the ribosomal RNA gene (*7*), followed by microbroth dilution susceptibility testing (*8*). All isolates were misidentified as *C. haemulonii* and *Rhodotorula glutinis* by Vitek-2 YST and API 20C AUX assays, respectively (Table).

**Table:** Identification and antifungal susceptibility results of 4 *Candida auris* isolates from 4 male patients with candidemia, South Africa, October 2012–October 2013*

Isolate ID	Patient age, y	Hospital unit	Vitek-2 YST†	API 20C AUX†	DNA sequence analysis‡	MIC								
						AMB	FLX	VRC	POS	ITC	5FC	CAS	MFG	AFG
208	85	High-care	*C. haemulonii*	*Rhodotorula glutinis*	*C. auris*	1	>256	0.5	0.03	0.12	0.12	0.25	0.06	0.25
209	60	Medical ICU	*C. haemulonii*	*R. glutinis*	*C. auris*	0.5	>256	1	0.06	0.12	0.12	0.12	0.06	0.12
224	73	Burn	*C. haemulonii*	*R. glutinis*	*C. auris*	1	>256	2	0.06	0.25	0.12	0.25	0.12	0.25
293	27	Trauma ICU	*C. haemulonii*	*R. glutinis*	*C. auris*	1	64	0.25	0.015	0.06	0.06	0.03	0.06	0.06

*AMB, amphotericin B; FLX, fluconazole; VRC, voriconazole; POS, posaconazole; ITC, itraconazole; 5FC, flucytosine; CAS, caspofungin; MFG, micafungin; AFG, anidulafungin. †bioMérieux, Marcy ľEtoile, France. ‡Sequence data for the 4 isolates have been deposited in GenBank, accession nos. KJ1236762–KJ126765 and KJ126758–KJ126761 for the internal transcribed spacer and D1/D2 regions, respectively.

Similar to the findings of Chowdhary et al., all isolates assimilated *N*-acetyl-glucosamine (*1*). With the use of the CBS-KNAW database, pairwise sequence alignment of ITS region showed 99% sequence homology to Kuwait isolates, and alignment of D1/D2 domain showed 98% homology to the Kuwait/India isolates (*9*). In a neighbor-joining phylogenetic tree based on ITS sequences, South Africa isolates formed a cluster with India and Kuwait isolates (Technical Appendix Figure).

Fluconazole MICs were high for all isolates (Table). Isolates 209 and 224 showed reduced voriconazole susceptibility with MICs of 1 μg/mL and 2 μg/mL, respectively, which is above the epidemiologic cutoff value for 11 *Candida* species (*10*). Isolates were susceptible to amphotericin B and echinocandins at low MICs Clinical data were available for 1 patient (Technical Appendix Table). Two *C. haemulonii* isolates were identified during laboratory-based sentinel surveillance for candidemia in South Africa; the ITS region of one isolate was sequenced and the isolate identified as C. auris (N.P. Govender, pers. comm.). In this study, *C. auris* was misidentified by routinely used tests and was accurately identified by sequencing, in keeping with previous findings (*1*,*3*,*4*,*6*).

Technical Appendix Phylogenetic relatedness of internal transcribed spacer region of the ribosomal RNA gene of *Candida auris* with closely related *Candida* species and clinical characteristics of a patient with candidemia caused by *C. auris*, South Africa.
