# Supplementary material for: Candida auris–Associated Candidemia, South Africa
Source: Emerg Infect Dis. 2014 Jul;20(7):1250–2. doi: 10.3201/eid2007.131765 (PMC4073876; doi:10.3201/eid2007.131765)
Supplement: Technical Appendix — Phylogenetic relatedness of internal transcribed spacer region of the ribosomal RNA gene of Candida auris with closely related Candida species and clinical characteristics of a patient with candidemia caused by C. auris, South Africa. [file 13-1765-Techapp-s1.pdf]

DOI: http://dx.doi.org/10.3201/eid2007.131765

Candida auris–Associated Candidemia, South Africa

Technical Appendix

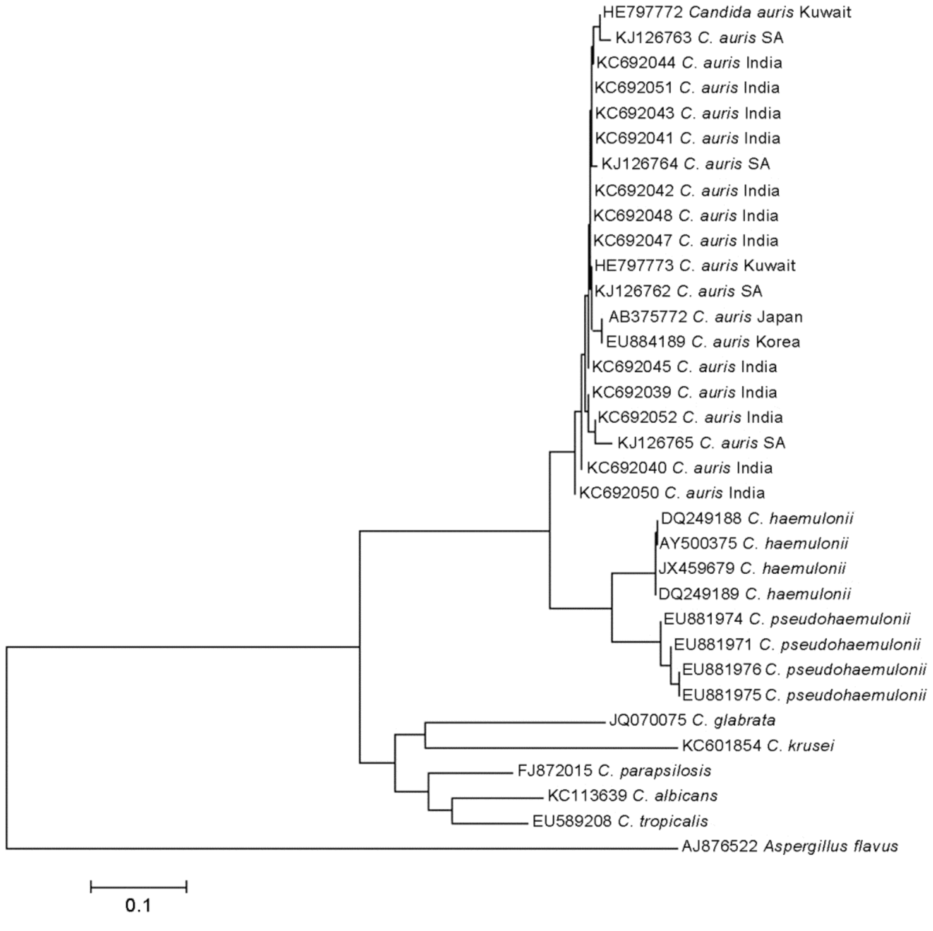

Technical Appendix Figure. Phylogenetic relatedness of internal transcribed spacer region of the ribosomal RNA gene of *Candida auris* with closely related *Candida* species. Scale bar indicates nucleotide substitutions per site.

Technical Appendix Table. Clinical characteristics of a 73-year-old male patient with candidemia caused by *Candida auris*, South Africa

Isolate ID	Risk factor	Antifungal treatment	Outcome
224	Referred to a public-sector specialist burn unit from a private-sector hospital<br>40% third-degree burns with inhalational injury; required débridement, skin grafts, and tracheostomy<br>In situ: central venous catheter/s, arterial line, urinary catheter<br>Mechanically ventilated<br>Multiple episodes of sepsis requiring broad-spectrum antimicrobial drugs, including β-lactams, colistin, linezolid, and vancomycin<br>Renal failure requiring hemodialysis	Amphotericin B deoxycholate (received only 1 dose)	Died 35 d after admission to hospital
